# Structural characterization of a GNAT family acetyltransferase from *Elizabethkingia anophelis* bound to acetyl-CoA reveals a new dimeric interface

**DOI:** 10.1038/s41598-020-79649-5

**Published:** 2021-01-14

**Authors:** P. Shirmast, S. M. Ghafoori, R. M. Irwin, J. Abendroth, S. J. Mayclin, D. D. Lorimer, Thomas E. Edwards, Jade K. Forwood

**Affiliations:** 1 School of Biomedical Sciences, Charles Sturt University, Wagga Wagga, NSW 2678 Australia; 2 Seattle Structural Genomics Center for Infectious Disease (SSGCID), Seattle, WA 98109 USA; 3 UCB, Bainbridge Island, Washington, 98110 USA

**Keywords:** Enzymes, Structural biology

## Abstract

General control non-repressible 5 (GCN5)-related N-acetyltransferases (GNATs) catalyse the acetylation of a diverse range of substrates, thereby orchestrating a variety of biological processes within prokaryotes and eukaryotes. GNAT enzymes can catalyze the transfer of an acetyl group from acetyl coenzyme A to substrates such as aminoglycoside antibiotics, amino acids, polyamines, peptides, vitamins, catecholamines, and large macromolecules including proteins. Although GNATs generally exhibit low to moderate sequence identity, they share a conserved catalytic fold and conserved structural motifs. In this current study we characterize the high-resolution X-ray crystallographic structure of a GNAT enzyme bound with acetyl-CoA from *Elizabethkingia anophelis*, an important multi-drug resistant bacterium. The tertiary structure is comprised of six α-helices and nine β-strands, and is similar with other GNATs. We identify a new and uncharacterized GNAT dimer interface, which is conserved in at least two other unpublished GNAT structures. This suggests that GNAT enzymes can form at least five different types of dimers, in addition to a range of other oligomers including trimer, tetramer, hexamer, and dodecamer assemblies. The high-resolution structure presented in this study is suitable for future in-silico docking and structure–activity relationship studies.

## Introduction

*Elizabethkingia anophelis* is a newly discovered gram negative bacterium, isolated from the midgut of *Anopheles gambiae* mosquito^1,2^. The bacterium is emerging as an important and opportunistic human pathogen, with capacity for causing neonatal meningitis and sepsis. Multi-drug resistance profiles against several antibiotics have been reported^3^, although the antimicrobial susceptibility and the resistance mechanism to antibiotics remain unresolved. Recent studies have reported antibiotic resistance to ampicillin, chloramphenicol, kanamycin, streptomycin and tetracycline^4,5^, while resistance against tetracycline, trimethoprim/sulfamethoxazole, and ciprofloxacin are of high concern since these antibiotics are first-line therapies for infections caused by this pathogen^6^. Infections caused by *E. anophelis* are generally hospital-acquired and include nosocomial pneumonia, bacteremia, sepsis, meningitis, skin and soft-tissue infection, urinary tract infection, and biliary tract infection in neonates and adults with underlying other diseases including malignancies and/or immunosuppression^7–9^. Several outbreaks of *E. anophelis* across many countries, has led to recent interest in discovering the underlying mechanisms of antibiotic resistance and possible new drug targets to overcome resistance^10–12^.

General control non-repressible 5 (GCN5)-related N-acetyltransferases (GNATs) are a large superfamily of enzymes, playing prominent roles across a large number of biological processes including aminoglycoside antibiotic resistance, transcription regulation, protein acetylation and stress reaction^13,14^. GNAT proteins possess a highly conserved catalytic fold, and can acetylate a wide range of substrates ranging from antibiotics to proteins^15,16^. The transfer of an acetyl group from acetyl-CoA (AcCoA) to substrates such as aminoglycoside antibiotics alter the fundamental characteristics of these molecules and can render them inactive^17,18^.

Despite the relatively low number of studies on bacterial protein acetylation, evidence is gathering to suggest that this post-translational modification occurs on many bacterial virulence factors and may play a key role in bacterial virulence^14^. Previous studies have demonstrated that GNAT enzymes of several bacteria are capable of acetylating different antibiotics^16,19,20^. *E. anophelis* strain 12012‐2PRCM has been reported to be resistant against aminoglycosides, β‐lactams, polypeptides, sulfonamides, chloramphenicols, quinolones, and tetracyclines^5^.

Previous structural characterizations of GNAT proteins have led to a consensus or conserved fold common to GNAT family members. Functional GNAT proteins generally contain six (or sometimes seven) β-strands and four α-helices arranged in a (β0)-β1-α1-α2-β2-β3-β4-α3-β5-α4-β6 topology^21^. These secondary structural elements comprise four conserved sequence motifs, motif C (β1, α1), motif D (β2-3-α2), motif A (β4-α3), B (β5-α4))^21^. The N-terminus is moderately well conserved while the C-terminal region varies considerably, in part, due to this region being responsible for substrate binding^22^. With the emergence of multi-drug resistance (MDR) in *E. anophelis*, here we determine the structure of an uncharacterized GNAT family acetyltransferase from this organism, which may provide a platform for *in-silico* fragment screening, drug-design, and/or structure–activity relationship studies.

## Materials and methods

### Cloning, expression, and purification

The gene for BAY10_3400 was amplified from genomic DNA and cloned into the expression vector BG1861 using ligation-independent cloning^23^. The expression vector provides a non-cleavable N-terminal His6-tag (SSGCID target ID ElanA.19303.a; UniProt ID A0A1T3E2H1). The enzyme was expressed in *E. coli* Rosetta BL21(DE3)R3 following standard SSGCID protocols, as described previously^24^. Purification was performed using Ni–NTA affinity and size exclusion chromatography following standard SSCID protocols^25^. Briefly, the bacterial cell pellet was suspended in a buffer comprised of 25 mM HEPES, 500 mM NaCl, 5% glycerol, 30 mM imidazole, 0.025% sodium azide, 0.5% CHAPS, 10 mM MgCl_2_, 1 mM TCEP, 250 µg/ml AEBSF, 0.05 µg/ml lysozyme pH 7.0). Cell lysis was undertaken by sonication, and the resulting extract clarified by centrifugation and passed over a HisTrap FF 5 ml column pre-equilibrated in 25 mM HEPES, 500 mM NaCl, 5% glycerol, 30 mM imidazole, 0.025% sodium azide, 1 mM TCEP pH 7.0. The column was washed with 20 column volumes of 25 mM HEPES, 500 mM NaCl, 5% glycerol, 30 mM imidazole, 0.025% sodium azide, 1 mM TCEP pH 7.0 to remove unbound proteins. The His-tagged protein was eluted with seven column volumes of a buffer comprised of 25 mM HEPES, 500 mM NaCl, 5% glycerol, 1 mM TCEP, 250 mM imidazole and 0.025% azide pH 7.0. The protein was further purified by size exclusion chromatography using a HiLoad 26/60 Superdex 75 preparative-grade column pre-equilibrated in 25 mM HEPES, 500 mM NaCl, 5% glycerol, 2 mM DTT, 0.025% azide pH 7.0, and calibrated using the GE Healthcare Calibration Kit (28-4038-41) with conalbumin, ovalbumin and ribonuclease A. Two protein peaks, corresponding to monomer and dimer species (molecular weights determined from the elution volume and a standard calibration curve) were pooled and analyzed by SDS–PAGE (see Supplementary Figure 1). The purified proteins were concentrated to 16 mg/ml (fractions from the dimer peak) and 36 mg/ml (fractions from the monomer peak) in 25 mM Hepes pH 7.5, 0.3 M NaCl, 1 mM DTT, 0.025% w/v sodium azide, 10% glycerol), flash frozen in liquid nitrogen and stored at − 80 °C.

### Crystallization, data collection, and structure determination

Crystallization trials were performed with apo protein at 16 mg/ml (from the sample obtained from the first elution peak) (see Supplementary Figure 1), and 36 mg/ml (from the sample obtained from the second elution peak) (see Supplementary Figure 1), using 96-well XJR crystallization trays (Rigaku Reagents) with 0.4 μl protein mixed with 0.4 μl reservoir, equilibrating against 80 µl reservoir solution. Crystallization conditions were searched for with sparse matrix screens JCSG + (Rigaku Reagents), CrystalScreen HT, Index HT (Hampton Research), and PACT (Molecular Dimensions). Crystallization trays were incubated at 285 K. Crystals were observed in all trays. The same crystals were obtained from both protein preparations, and a crystal formed in 100 mM Tris/HCl pH 5.5, 2 M ammonium sulfate, and 5 mM acetyl-CoA) from the 16/ml sample was cryo-protected with a solution of reservoir with 25% ethylene glycerol, and vitrified in liquid nitrogen. Diffraction data were collected at the Advanced Photon Source Life Sciences Collaborative Access Team (APS LS-CAT) beamline 21 ID-G equipped with a Rayonic MX-300 CCD detector at a wavelength of 0.97856 Å. The same shaped crystals grown from the 36 ml/ml sample were not diffracted due to time limitations at the Synchrotron. Data sets were reduced with the XDS package^26^. Diffraction data are summarized in Table 1.

**Table 1:** Data collection, refinement and structure quality.

Parameter	Value
PDB ID	6AO7
Space group	*P*6_5_22
**Unit-cell parameters**	
*a* (Å)	67.16
*b* (Å)	67.16
*c* (Å)	134.21
α = β (°)	90
γ (°)	120
Matthews coefficient (Å^3^Da^−1^)	2.33
Solvent content (%)	47.25
Resolution range (Å)	50–1.85 (1.90–1.85)^a^
Mean I/σ(I)	35.45 (4.88)
No. of observed reflections	15,998 (1150)
Completeness (%)	99.6 (100)
Multiplicity	13.8 (14.3)
R_merge_	0.038 (0.515)
**Refinement**	
No. of used reflections	15,975
R_work_ (%)	0.200
R_free_ (%)	0.244
Mean B factor (Å^2^)	48.0
RMSD bonds (Å)	0.007
RMSD angles (°)	0.901
**Model validation**^**b**^	
MolProbity	
Clash score, all atoms	2.84
Ramachandran favored (%)	98.7
Ramachandran outliers (%)	0
MolProbity score	1.23

^a^Value in parenthesis are statistics for the highest resolution shell.

^b^Values calculated using Molprobity.

The structure of the enzyme was solved by molecular replacement using the program Molrep^27^ from the CCP4 package^28^, with PDB entry 1YVK as the search model. The initial model was extended with ARP/wARP^29^. Manual model building was performed using Coot^30^, and the structure was refined in reciprocal space with Phenix^31^. The coordinates and structure factors of the apo structure were deposited in the PDB with accession code 6AO7.

## Results and discussion

### Protein expression and structure determination

To determine the crystallographic structure of an uncharacterized GNAT from *E. anophelis* and allow the identification of key motifs and structural hallmarks common to GNAT family members, we first cloned the gene and recombinantly expressed the protein as a 6-His fusion (see “Materials and methods”). Following purification of the His-tagged protein by affinity purification, the protein was further purified by size exclusion chromatography, yielding two peaks, corresponding to monomer and dimer species (Supplementary Figure 1). Analysis by SDS-PAGE confirmed that the same protein was present in both peaks (Supplementary Figure 1), and each peak was concentrated to 16 mg/ml (peak 1) and 36 mg/ml (peak 2) (Supplementary Figure 1). Crystallisation trials were performed with both protein preparations, and despite minor protein contaminations (Supplementary Figure 1), the same crystal morphology was obtained with both preparations in 100 mM Tris/HCl pH 5.5, 2 M ammonium sulfate, and 5 mM acetyl-CoA. Crystals grown from the protein preparation at 16 mg/ml (obtained from the dimer species) diffracted to 1.85 Å. Diffraction data were indexed and integrated in the space group *P*6_5_22, with unit cell parameters *a* = 67.16 Å, *b* = 67.16 Å, *c* = 134.21 Å, α = β = 90°, γ = 120°. The structure was solved using molecular replacement in Molrep^27^ using chain A of PDB model 1YVK. One molecule was placed in the asymmetric unit and following model rebuilding in COOT^30^ and refinement in Phenix^31^, a final model was produced with an R_cryst_ and R_free_ of 0.195 and 0.244 respectively. With the exception of the N-terminal methionine, all amino acid residues were modelled, and coordinates and associated data files were deposited to the Protein Data Bank and issued the PDB ID 6AO7. The reflection data, model, and refinement statistics are summarized in Table 1.

### Structural characterization reveals the putative GNAT from *Elizabethkingia anophelis* contains the motifs and hallmarks for a GNAT

**Figure 1:**
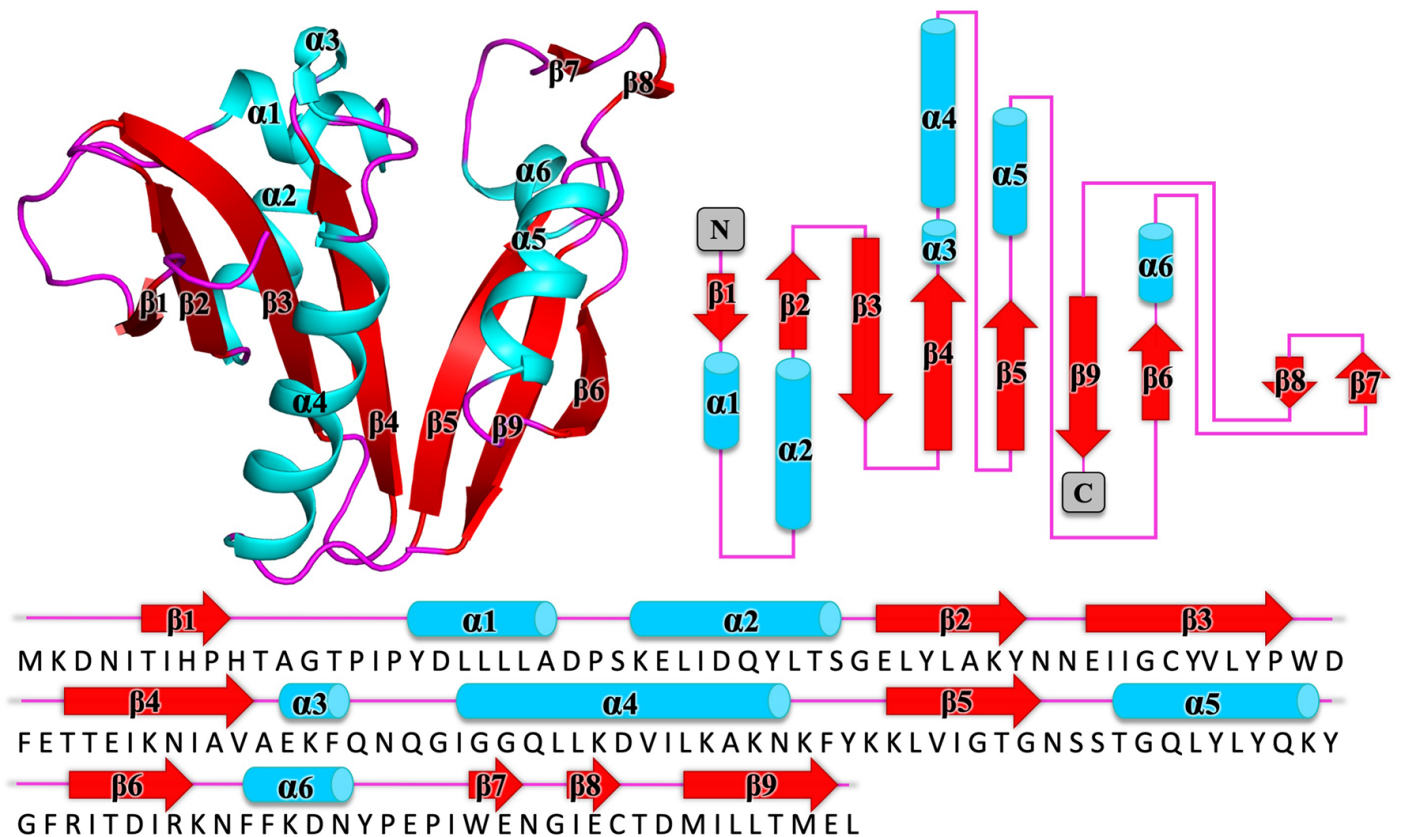
Tertiary structure the GNAT acetyltransferase from *Elizabethkingia anophelis*. Top left, shown in cartoon format, α-helices, β-strands, and loops are shown in cyan, red, and magenta, respectively. Top right, topology map of the GNAT acetyltransferase from *Elizabethkingia anophelis* with α-helices shown as cylinders colored in cyan, β-strands are presented as red arrows, and loops are shown with magenta. Bottom panel, sequence of the GNAT acetyltransferase from *Elizabethkingia anophelis* spanning these structural elements.

The protein was structured as an α/β protein and comprised of six α-helices and nine β-strands with a topology of β1-α1-α2-β2-β3-β4-*α3*-α4-β5-α5-β6-*α6*-*β7*-*β8*-β9 (Fig. 1). The core follows the conserved topology pattern of a typical GNAT family member, β1-α1-α2-β2-β3-β4-α3-β5-α4-β6, with additional secondary structural elements highlighted in italics. The structure exhibits two β-sheets comprised of β-strands 1-4, and β-strands 5, 6, and 9, splayed to create a V-shape (Fig. 1). Stabilization of the splayed β-sheets is mediated through H-bonding between Thr^63^ and Ile^65^ on β-strand 4, and Val^100^ on β-strand 5.

**Figure 2:**
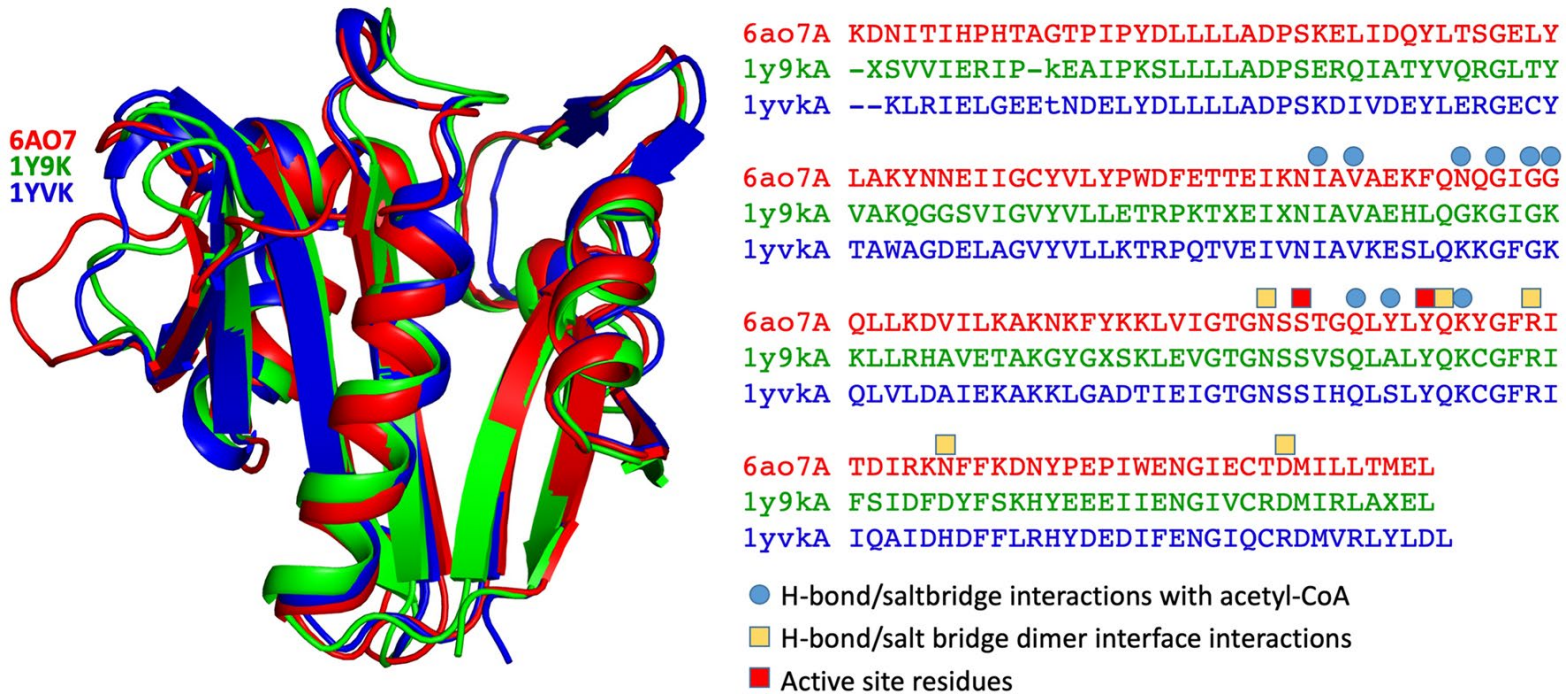
Tertiary structure the GNAT acetyltransferase from *Elizabethkingia anophelis* (shown in cartoon format in red), superimposed with the two most closely related structures identified from DALI (1Y9K in green, and 1YVK in blue). The alignment of these corresponding sequences are presented on the right, with active site residues highlighted with a red box, residues involved in dimer interface bonding in yellow boxes, and residues involved in acetyl-CoA binding in blue circles.

The GNAT has not been characterized previously, and we therefore performed both BLAST and DALI searches to determine proteins with similar sequence and structural features. A sequence homology search (excluding Elizabethkingia (taxid: 308865)), revealed the most closely related proteins to be GNAT family members from *Runella zeae* (55% sequence identity with 100% coverage), *Microscillaceae bacterium* (60% identity with 90% coverage), and *Caenibacillus caldisaponilyticus* (59% identity with 90% coverage). Within the top 20 Blast results, 3 GNAT proteins were aminoglycoside 6′-N-acetyltransferases: *Chitinophaga eiseniae* (53% identity with 98% coverage)*, Pedobacter nutrimenti* (59% identity and 89% coverage), and *Rhabdobacter roseus* (50% identity and 98% coverage) (Supplementary Table 1). Moreover, docking of kanamycin produced a structural model within the active site and no steric clashes (Supplementary Figure 2), however further experimentation is required to confirm the substrate. Since GNAT proteins often exhibit low sequence identity but high structural homology^15^, we also performed a DALI search. We found only one protein with a rmsd value of less than 1 Å, PDB 1y9k (0.9 Cα rmsd, 48% seq id; no primary citation), solved by a structural genomics consortium that remains to be published. The next most similar protein was 1yvk (1.4 Cα rmsd, 49% seq id; no primary citation), and this was also solved by a structural genomics group and remains unpublished. The sequence identity between 1y9k and 1yvk is 59% (the top10 Dali results and a sequence based structural alignment are presented in Supplementary Table 2 and Supplementary Figure 3 respectively). Interestingly, 1y9k is predicted to be a dimer, while 1vyk is predicted to be a tetramer (discussed below). All other PDB entries have a sequence identity of less than 20%, and Cα rmsd greater than 2. Structural superposition of these structures, together with a structure-based sequence alignment is presented in Fig. 2.

### Structural analysis of the acetyl-CoA binding site

**Figure 3:**
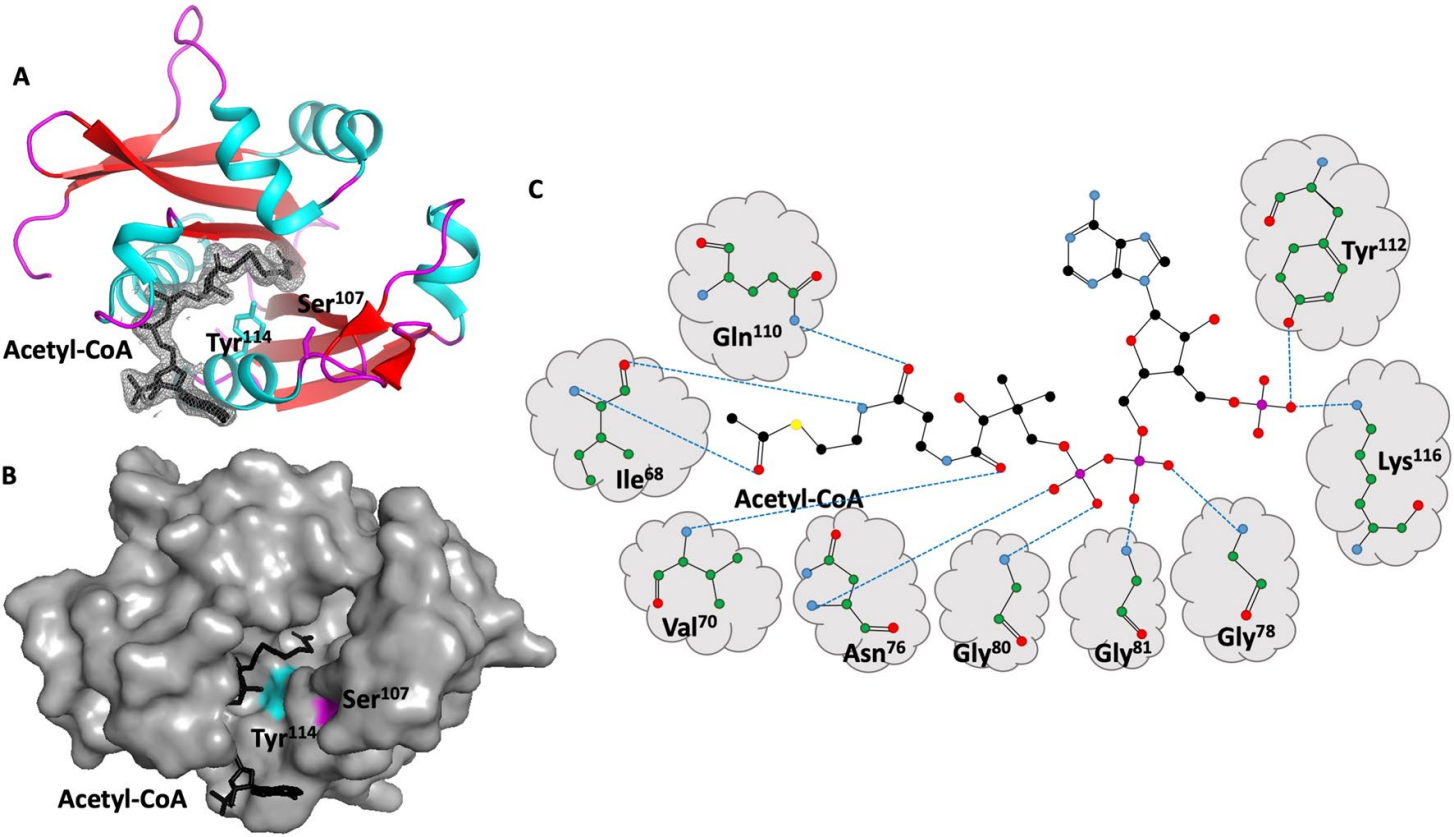
Acetyl-CoA binding site depicted within the tertiary structure the GNAT acetyltransferase from *Elizabethkingia anophelis.* Top left, shown in cartoon format, α-helices, β-strands, and loops are shown in cyan, red, and magenta, respectively. Acetyl-CoA is depicted in stick mode in black, with associated electron density map (2Fo-Fc) contoured at 1.0σ. The side chains of active site residues Tyr114 and Ser107 are depicted in stick mode. Bottom left, the same structure as above, but shown in surface view and coloured grey to depict the binding cleft. The location of Tyr114 and Ser107 are coloured cyan and magenta respectively. Right panel, schematic of hydrogen bond interactions between GNAT acetyltransferase residues and acetyl-CoA.

Our structure contained a well-ordered acetyl-CoA molecule bound in the active site (Fig. 3AB). The interface between acetyl-CoA buries 593 Å^2^ of surface area and mediated by 10 hydrogen bonds. Key residues involved in these hydrogen bond interactions include Ile^68^, Val^70^, Asn^76^, Gly^78^, Gly^80^, Gly^81^, Gln^110^, Tyr^112^, and Lys^116^ (Fig. 3C), and specific interactions are summarized in Table 2. Based on the position of the acetyl-CoA in the structure and well characterized kinetic studies of GNAT enzymes, the most likely active residues are Ser^107^ and Try^114^. Most GNATs contain either a Glu, Asp or Ser near the active site, which serves to act as a general base by extracting a proton from the substrate^15,32–34^. Nucleophilic attack on acetyl-CoA leads to the creation of a transient zwitterionic tetrahedral intermediate, and receival of a proton from a general acid (usually Tyr or Ser)^15,35^. Approximately 62% of GNATs have been reported to contain a conserved Tyr as a general acid to initiate the catalysis, and the positioning of Tyr^114^ from our structural analysis is consistent with this^36^. Both the acetyl-CoA residues involved in H-bonding, and active site residues are shown in Fig. 2.

**Table 2:** Hydrogen bond interactions between GNAT acetyltransferase from *Elizabethkingia anophelis* and acetyl-CoA.

Bonding atom/residue			Distance	Acetyl-CoA (ACO)		
N	ILE	68	3.12	O	ACO	201
O	ILE	68	2.81	N4P	ACO	201
N	VAL	70	2.76	O9P	ACO	201
N	ASN	76	3.13	O5A	ACO	201
N	GLY	78	2.79	O1A	ACO	201
N	GLY	80	2.86	O4A	ACO	201
N	GLY	81	3.04	O2A	ACO	201
NE2	GLN	110	2.95	O5P	ACO	201
OH	TYR	112	2.56	O9A	ACO	201
NZ	LYS	116	2.51	O9A	ACO	201

### Quaternary structure

**Figure 4:**
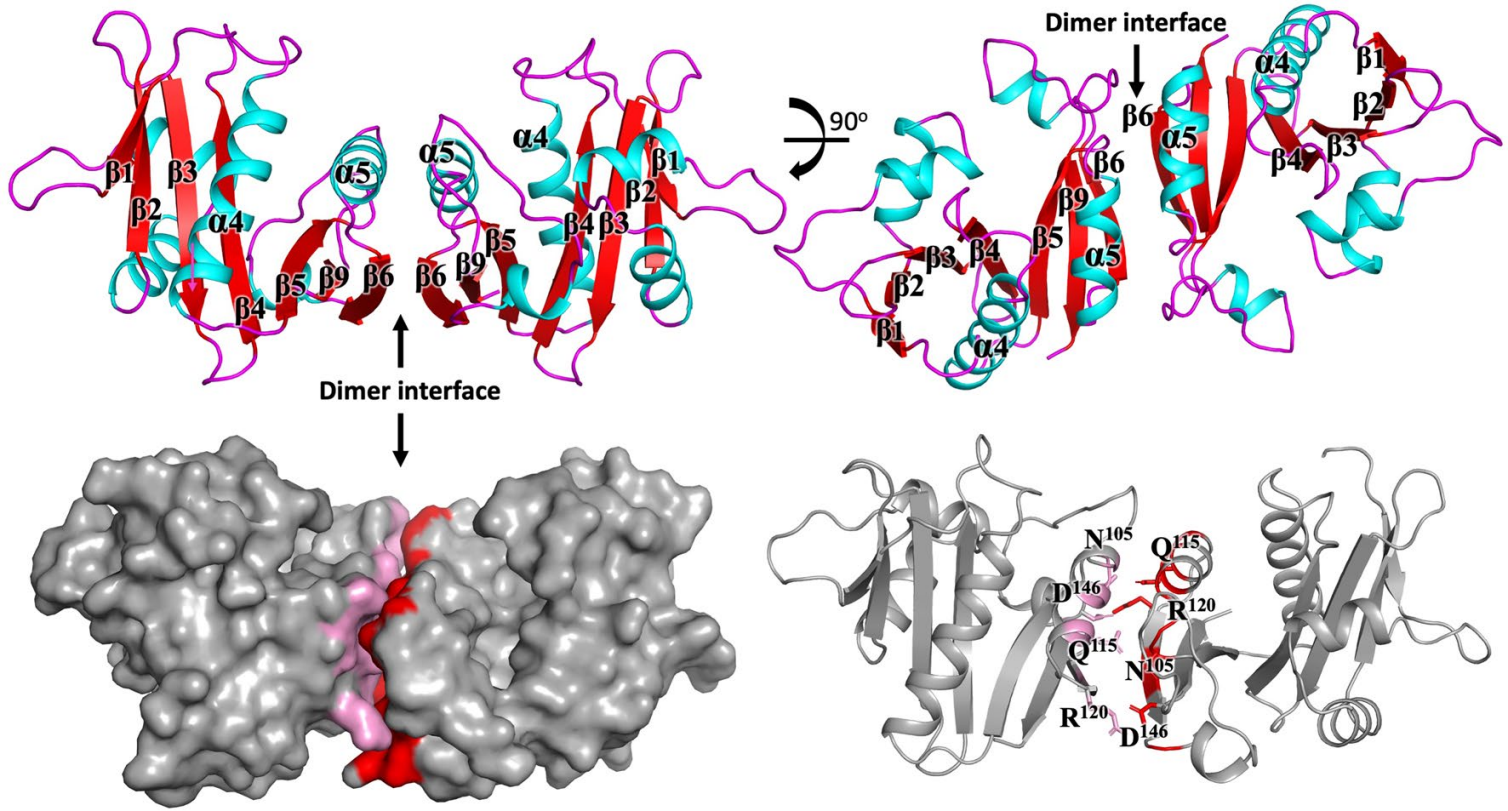
The structure of the GNAT acetyltransferase from *Elizabethkingia anophelis* exhibits a significant dimer interface*.* The secondary structural elements mediating the dimer interface are presented in the left panels. Residues within α-helix five are depicted in the right panel and pack form an array of hydrogen bond and salt bridge interactions summarized in Tables 3 and 4.

GNATs adopt a wide variety of assemblies, ranging from monomers, dimers, trimers, tetramers, hexamers, and dodecameric (double ring) structures^37–44^. Analysis of the interfaces present in the crystal using PISA^45^ suggests the protein is most likely to be a dimer (Fig. 4).

**Figure 5:**
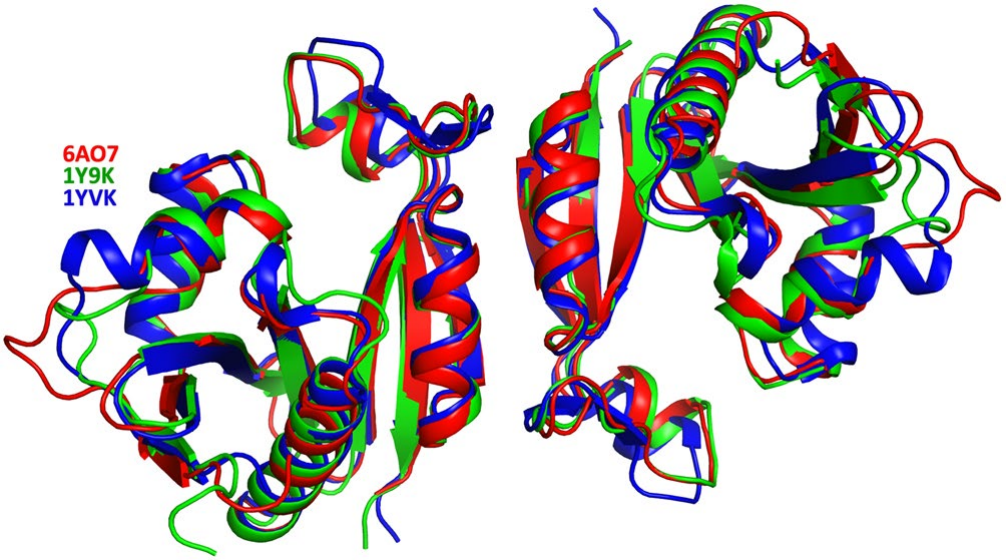
The dimer interface observed in the GNAT acetyltransferase from *Elizabethkingia anophelis* is conserved in two other unpublished structures. Superposition of the two structures (PDBs 1Y9K (green) and 1YVK (blue), both unpublished), demonstrate a conserved dimer interface.

**Figure 6:**
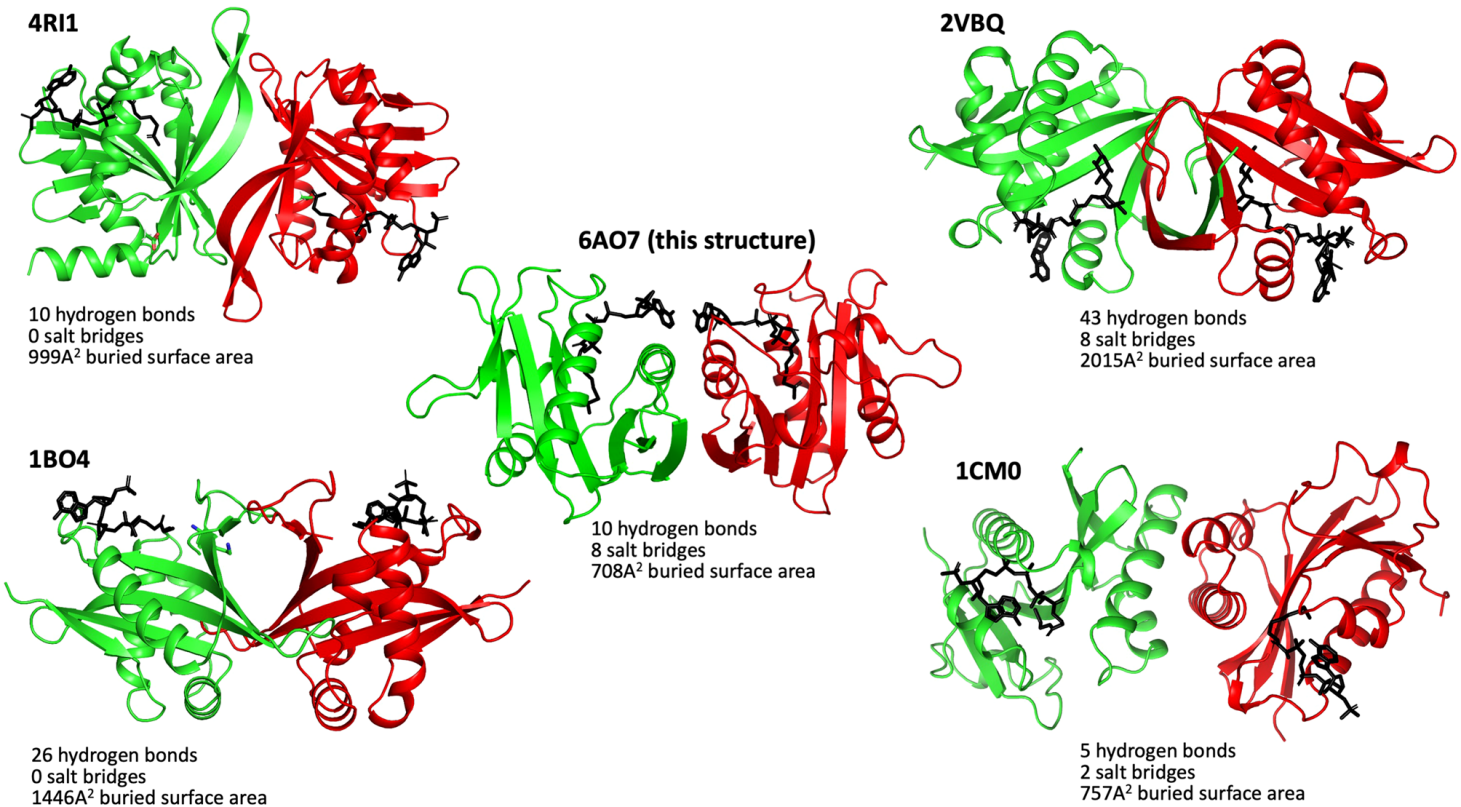
GNATs form a range of different dimers. Presented here are the 5 (including this dimer) different dimer structures. The PDB codes 4RI1, 2VBQ, 1BO4 and 1CM0, representing each different dimer are shown below each dimeric structure.

To test whether this interface is conserved in the most closely related GNATs, we examined whether the same dimer structures were present from proteins of different crystal space groups and distinctly different sequences. We found that the same dimer structure is present in both of the crystal structures of 1YVK and 1Y9K (Fig. 5). Since these sequences are distinctly different, and the crystal space groups are unrelated in all three structures, this suggests that these dimer interfaces are unlikely to be a crystal artefact. In this assembly configuration, the two monomers bury a surface area of 1464 Å, and mediated by ten hydrogen bonds (Table 3) and eight salt bridges (Table 4). Key interactions at the dimer interface include Arg^120^ forming a salt bridge interaction with Asp^146^, and Gln^115^ hydrogen bonding with Asn^105^. Dimers are the most common GNAT assembly, and there are four different types of dimers that have been described in the literature^21^. One form involves the C-terminal β-strands interacting to form a continuous β-sheet^15,46–49^. A second form involves two monomers exchanging their C-terminal β-strands (Eg ScHpa2, SeAAC(6′)-Iy and ScGNA1^39,50,51^. A third form involves two monomers forming a β-barrel (eg SmAAC(3)-Ia^52^, while the fourth type is formed through a four-helical bundle (e.g. pPCAF)^53^. The dimer interface found in our structure is distinctly different to any other dimer described previously (Fig. 6), and is identical in at least two other GNATs suggesting this dimer interface is conserved.

**Table 3:** Hydrogen bonding at the dimer interface.

Interface on chain A	Distance (Å)	Interface on chain B
A:GLN 115[NE2]	2.92	B:ASN 105[O]
A:ASN 105[ND2]	2.76	B:GLN 115[OE1]
A:ARG 120[NH2]	3.13	B:ASN 127[OD1]
A:ARG 120[NE]	2.98	B:ASP 146[OD1]
A:ARG 120[NH2]	2.75	B:ASP 146[OD2]
A:ASN 105[O]	2.92	B:GLN 115[NE2]
A:GLN 115[OE1]	2.76	B:ASN 105[ND2]
A:ASN 127[OD1]	3.13	B:ARG 120[NH2]
A:ASP 146[OD1]	2.98	B:ARG 120[NE]
A:ASP 146[OD2]	2.75	B:ARG 120[NH2]

**Table 4:** Salt bridge interactions at the dimer interface.

Interface on chain A	Distance (Å)	Interface on chain B
A:ARG 120[NH2]	3.36	B:ASP 146[OD1]
A:ARG 120[NE]	2.98	B:ASP 146[OD1]
A:ARG 120[NH2]	2.75	B:ASP 146[OD2]
A:ARG 120[NE ]	3.84	B:ASP 146[OD2]
A:ASP 146[OD1]	2.98	B:ARG 120[NE]
A:ASP 146[OD1]	3.36	B:ARG 120[NH2]
A:ASP 146[OD2]	3.84	B:ARG 120[NE]
A:ASP 146[OD2]	2.75	B:ARG 120[NH2]

## Conclusion

Here, we describe the structure of a GNAT family member from *E. anophelis*. Structural analysis suggests that the protein exhibits the hallmarks typical of a GNAT fold. Structural and sequence-based alignments suggest that this acetyltransferase may be a possible aminoglycoside 6′-N-acetyltransferase, however this needs to be determined experimentally. Residues involved in acetyl-CoA binding have been identified and are conserved in closely related GNATs. We identify a new type of dimeric interface, and this is conserved in at least two other structures that have been deposited to the PDB, but remain unpublished. The oligomerization of GNATs vary range from dimers through to dodecaomers, and oligomerization is important for function^15^. Thus characterization of new Gcn5-related N-acetyltransferases has the potential to expand our understanding of how these enzymes carry out acetylation of a wide range of substrates, and high-resolution structural elucidation may offer new opportunities for biotechnological applications and drug design.

## Supplementary Information

Supplementary Information.
