# Supplementary material for: Structural characterization of a GNAT family acetyltransferase from Elizabethkingia anophelis bound to acetyl-CoA reveals a new dimeric interface
Source: Sci Rep. 2021 Jan 14;11:1274. doi: 10.1038/s41598-020-79649-5 (PMC7809356; doi:10.1038/s41598-020-79649-5)
Supplement: Supplementary file 1 — Supplementary Information. [file 41598_2020_79649_MOESM1_ESM.pdf]

Structural characterization of a GNAT family acetyltransferase from *Elizabethkingia anophelis* bound to acetyl-CoA reveals a new dimeric interface

Shirmast, P.[1], Ghafoori, SM.[1], Irwin, R.M[2,3], Abendroth, J.[2,3], Mayclin, S.J. [2,3], Lorimer, D.D. [2,3], Edwards, T.E. [2,3,*], Forwood J.K.[1,*].

Supplementary Figure 1. Purification of the GNAT family acetyltransferase from Elizabethkingia anopheles. Left panel, size exclusion elution profile showing the presence of a dimer and monomer species, together with a calibration curve associated with the size exclusion column. Right panel, SDS-PAGE analysis of the fractions obtained from the size exclusion column. Both the dimer and monomer were collected and used for crystallisation. Both produced same crystal forms.

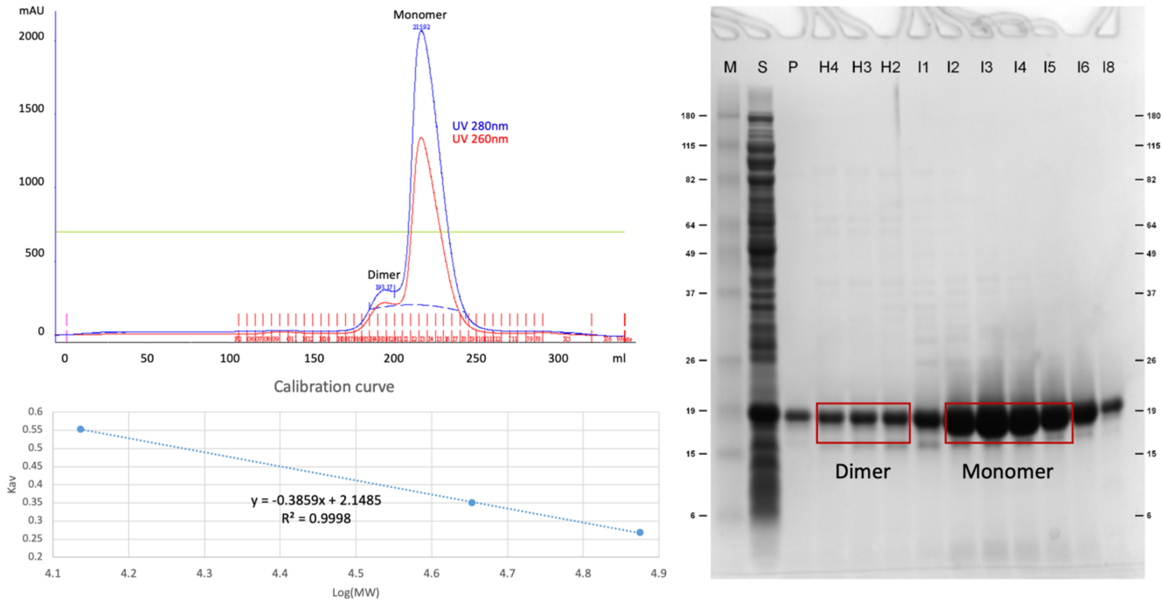

Supplementary Figure 2. Docking of kanamycin into the GNAT presented in this study using SWISSDOCK, highlighting the position of the active site in relation to dimer interface, acetyl-CoA, and the substrate cavity. Additional studies are required to determine the precise substrate.

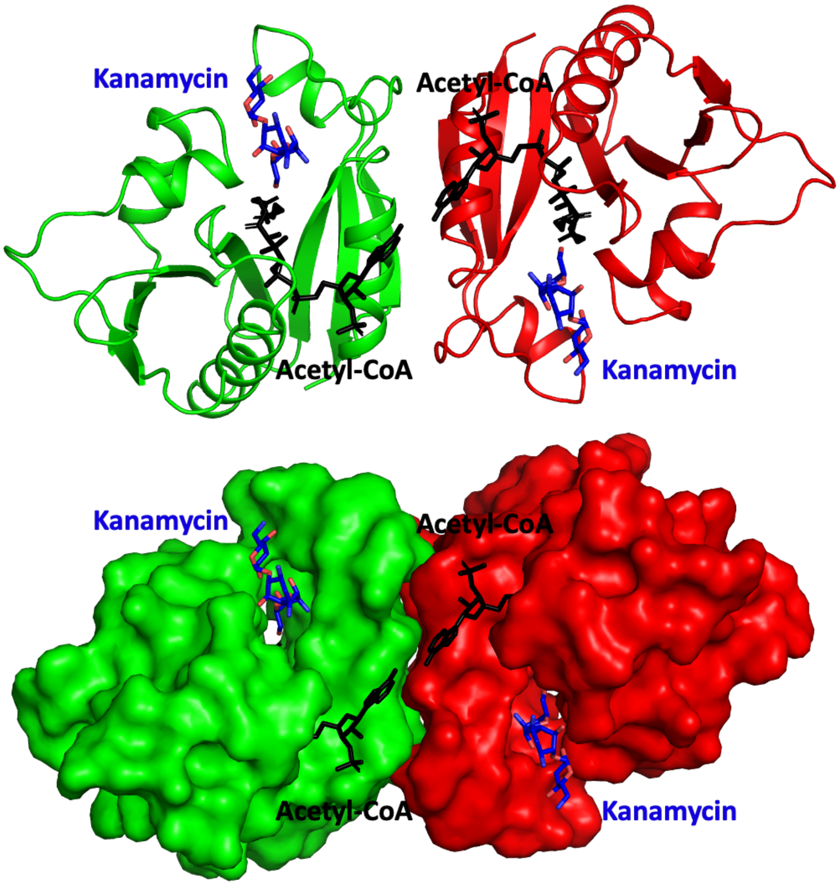

Supplementary Figure 3. Structure-based sequence alignment of the top 10 DALI results. PDB codes are indicated on the left. Secondary structure annotations are presented below, with loop (L), helix (H), and strand (E).

```
6ao7A K--DNITIHPHT--AGT-PI--PYDLL-L-LA---D-PSKE--LIDQYLTS-G-----ELYLAKY-----NNEIIGCYVLYPWD----
6ao7A K--DNITIHPHT--AGT-PI--PYDLL-L-LA---D-PSKE--LIDQYLTS-G-----ELYLAKY-----NNEIIGCYVLYPWD----
1y9kA ---XSVVIERIP---kE-AI--PKSLL-L-LA---D-PSER--QIATYVQR-G-----LTYVAKQ-----GGSVIGVYVLLETR----
1yvkA ----KLRIELGE--EtN-DE--LYDLL-L-LA---D-PSKD--IVDEYLER-G-----ECYTAWA-----GDELAGVYVLLKTR----
2cnmA ----MNTISILS---TT-DLpaAWQIE-Q-RahafP-WSEK--TFFGNQGErY-----LNLKLTA-----DDRMAAFAITQV-V----
6wfnA ---SRIELGDVT--PHNiKQ--LKRLN-QvIF---P-VSYN-dKFYKDVLE-V---geLAKLAYF-----NDIAVGAVCCRVDH-sqn
4pv6A inaVAGTIREFS---PK-DIesVYRIA-QtSL---T-EYYTqaLILDLHRE-W---peSFMVYTV-----AGSVVGFIVGSKYS----
6wfgA ----RIELGDVT--PHNiKQ--LKRLNqV-IF---P-VSYN-dKFYKDVLE-V---geLAKLAYF-----NDIAVGAVCCRVDH-sqn
6ygaA ---XEIVYKPLDirNEE-QFasIKKLI-D-AD---LsEPYSiyVYRYFLNQ-W---peLTYIAVDnksgtPNIPIGCIVCKXDPhrnv
4x5kA --gSRIELGDVT--PHNiKQ--LKRLN-QvIF---P-VSYN----DKFYKD-VlevgeLAKLAYF-----NDIAVGAVCCRVDH-sqn
2ob0A skgSRIELGDVT--PHNiKQ--LKRLN-QvIF---P-VSYN-dkFYKDVLE-V---geLAKLAYF-----NDIAVGAVCCRVDH-sqn

6ao7A L--LLLEEEELL--LLL-LL--LHHHH-H-HH---L-LLHH--HHHHHHHH-L-----EEEEEEE-----LLEEEEEEEEEELL----
6ao7A L--LLLEEEELL--LLL-LL--LHHHH-H-HH---L-LLHH--HHHHHHHH-L-----EEEEEEE-----LLEEEEEEEEEELL----
1y9kA ---LLLEEEEEL---hH-HL--LHHHH-H-HH---L-LLHH--HHHHHHHH-L-----EEEEEEL-----LLLEEEEEEEEELL----
1yvkA ----LLEEEELL--LlL-HH--HHHHH-H-HH---L-LLHH--HHHHHHHH-L-----EEEEEEE-----LLEEEEEEEEEELL----
2cnmA ----LEEEEELL---HH-HHhhHHHHH-H-HhlllL-LLHH--HHHHLLLLlL-----LLEEEEE-----LLEEEEEEEEEE-E----
6wfnA ---LLEEEEELL--LLLhHH--HHHHH-HhHL---L-LLLL-hHHHHHHLL-L---hhHEEEEEE-----LLEEEEEEEEEEEE-ell
4pv6A lllLLLEEEELL---HH-HHhhHHHHH-HhHL---L-LLLLhhHHHHHHHH-L---hhHLEEEEE-----LLEEEEEEEEEELL----
6wfgA ----LEEEEELL--LLLhHH--HHHHHhH-HL---L-LLLL-hHHHHHHHH-L---hhHEEEEEE-----LLEEEEEEEEEEEE-ell
6ygaA ---LLEEEEELLllLHH-HHhhHHHHH-H-HH---LlLLLLhhHHHHHHLL-L---hhHEEEEEElllllLLLEEEEEEEEEEEelll
4x5kA --lLLEEEEELL--LLLhHH--HHHHH-HhHL---L-LLLL----HHHHHH-HhhlhhHEEEEEE-----LLLEEEEEEEEEEE-ell
2ob0A lllLLEEEEELL--LLLhHH--HHHHH-HhHL---L-LLLL-hhHHHHHLL-L---hhHEEEEEE-----LLEEEEEEEEEEEE-ell

6ao7A FETTEIKNIAVAEKFQNQGIGGQLLKDVILKAKNK-FYKKLVIGTGNSSTGQLYLYQKYGFRITDIRKNFFKDNYPEPIWENGIECTDMILLTMEL-----
6ao7A FETTEIKNIAVAEKFQNQGIGGQLLKDVILKAKNK-FYKKLVIGTGNSSTGQLYLYQKYGFRITDIRKNFFKDNYPEPIWENGIECTDMILLTMEL-----
1y9kA PKTXEIXNIAVAEHLQGKGIGKKLLRHAVETAKGY-GXSKLEVGTGNSSVSQLALYQKCGFRIFSIDFDYFSKHYEEEIIENGIVCRDXIRLAXEL----n
1yvkA PQTVEIVNIAVKESLQKKGFGKQLVLDAIEKAKKL-GADTIEIGTGNSSIHQLSLYQKCGFRIQAIDHDFFLRHYDEDIFENGIQCRDXVRLYLDL----l
2cnmA LDEATLFNIAVDPDFQRRGLGRMLLEHLIDELETR-GVVTLWLEVRASNAAAIALYESLGFNEATIRRNYYP--TAQG-------hEDAIIMALPIsmklh
6wfnA QKRLYIMTLGCLAPYRRLGIGTKMLNHVLNICEKDgTFDNIYLHVQISNESAIDFYRKFGFEIIETKKNYY-----KRIE-----PADAHVLQKNL-----
4pv6A RTEARILLFAVDERFRRMGVGSALMDAFLSLCREQ-NMLSVRLEVRTDNDEAIRFYKKYGFVITAMLPNYYSD------------sSNAYTMWRIVlehhh
6wfgA QKRLYIMTLGCLAPYRRLGIGTKMLNHVLNICEKDgTFDNIYLHVQISNESAIDFYRKFGFEIIETKKNYY-----KRIE-----PADAHVLQKNL-----
6ygaA RLRGYIGXLAVESTYRGHGIAKKLVEIAIDKXQRE-HCDEIXLETEVENSAALNLYEGXGFIRXKRXFRYYL------------NEGDAFKLILPL----t
4x5kA QKRLYIMTLGCLAPYRRLGIGTKMLNHVLNICEKDgTFDNIYLHVQISNESAIDFYRKFGFEIIETKKNYY-----KRIE-----PADAHVLQKNL----k
2ob0A QKRLYIXTLGCLAPYRRLGIGTKXLNHVLNICEKDgTFDNIYLHVQISNESAIDFYRKFGFEIIETKKNYY----KRIE------PADAHVLQKNL-----

6ao7A LLEEEEEEEEEELHHHLLLLHHHHHHHHHHHHHHL-LLLEEEEEEELLLHHHHHHHHHLLLEEEEEELLHHHHHLLLLLEELLEELLLEEEEEEEL-----
6ao7A LLEEEEEEEEEELHHHLLLLHHHHHHHHHHHHHHL-LLLEEEEEEELLLHHHHHHHHHLLLEEEEEELLHHHHHLLLLLEELLEELLLEEEEEEEL-----
1y9kA LLEEEEEEEEEELHHHLLLLHHHHHHHHHHHHHHL-LLLEEEEEEELLLHHHHHHHHHLLLEEEEEELLHHHHHLLLLEEELLEEELLEEEEEEEL----l
1yvkA LLEEEEEEEEEELHHHLLLLHHHHHHHHHHHHHHL-LLLEEEEEEELLLHHHHHHHHHLLLEEEEEELLHHHHLLLLLEEELLEEELLEEEEEEEL----l
2cnmA LLEEEEEEEEEELHHHLLLLHHHHHHHHHHHHHHL-LLLEEEEEEELLLHHHHHHHHHHLLEEEEEEEEEEE--ELLE-------eEEEEEEEEELlllll
6wfnA EEEEEEEEEEEELHHHLLLLHHHHHHHHHHHHHHHlLLLEEEEEEELLLHHHHHHHHHLLLLEEEEELLLL-----LLLL-----LLLEEEEEEEL-----
4pv6A LLEEEEEEEEEELHHHLLLLHHHHHHHHHHHHHHL-LLLEEEEEEELLLHHHHHHHHHLLLEEEEEELLLLLL------------lLLEEEEEEELlllll
6wfgA EEEEEEEEEEEELHHHLLLLHHHHHHHHHHHHHHHlLLLEEEEEEELLLHHHHHHHHHLLLLEEEEELLLL-----LLLL-----LLLEEEEEEEL-----
6ygaA EEEEEEEEEEEELHHHLLLLHHHHHHHHHHHHHHL-LLLEEEEEEELLLHHHHHHHHHLLLLEEEEELLLLH------------HHLLEEEEEEEL----l
4x5kA EEEEEEEEEEEELHHHLLLLHHHHHHHHHHHHLLLlLLLEEEEEEELLLHHHHHHHHHLLLLEEEEELLLL-----LLLL-----LLLEEEEEEEL----l
2ob0A EEEEEEEEEEEELHHHLLLLHHHHHHHHHHHHHHHlLLLEEEEEEELLLHHHHHHHHHLLLLEEEEELLLL----LLLL------LLLEEEEEEEL-----
```

Supplementary Table 1. Top 20 Blast results performed using the sequence from UniProt ID A0A1T3E2H1 and excluding Elizabethkingia (taxid:308865). Within the top 20 Blast results, 3 GNAT proteins were aminoglycoside 6'-N-acetyltransferases, highlighted in bold: Chitinophaga eiseniae (53% identity with 98% coverage), Pedobacter nutrimenti (59% identity and 89% coverage), and Rhabdobacter roseus (50% identity and 98% coverage).

Description	Max Score	Total Score	Query Cover	E value	Per. ident	Accession
GNAT family N-acetyltransferase [Runella zeae]	170	170	100%	3.00E-51	54.55	WP_037327508.1
GNAT family N-acetyltransferase [Microscillaceae bacterium]	169	169	90%	1.00E-50	60.43	HAI77368.1
GNAT family N-acetyltransferase [Caenibacillus caldisaponilyticus]	167	167	90%	3.00E-50	58.57	WP_077616020.1
GNAT family N-acetyltransferase [Sporocytophaga myxococcoides]	167	167	90%	4.00E-50	58.57	WP_028980556.1
**Aminoglycoside 6'-N-acetyltransferase I [Chitinophaga eiseniae]**	167	167	98%	6.00E-50	52.63	SKA33870.1
GNAT family N-acetyltransferase [Emticicia sp. 17J42-9]	166	166	96%	1.00E-49	54.73	WP_130020866.1
GNAT family N-acetyltransferase [Fictibacillus gelatini]	162	162	89%	6.00E-48	55.07	WP_026679390.1
**Aminoglycoside 6'-N-acetyltransferase I [Pedobacter nutrimenti]**	160	160	89%	1.00E-47	58.7	PYF75880.1
GNAT family N-acetyltransferase [Jeotgalibaca sp. PTS2502]	160	160	89%	3.00E-47	57.97	WP_076768550.1
GNAT family N-acetyltransferase [Bacillus yapensis]	159	159	90%	4.00E-47	54.29	RTR35708.1
GNAT family N-acetyltransferase [Solitalea canadensis]	160	160	98%	4.00E-47	54.3	WP_014680410.1
GNAT family N-acetyltransferase [Filimonas effusa]	159	159	98%	6.00E-47	49.67	WP_129005358.1
GNAT family N-acetyltransferase [Rhodocytophaga sp. 172606-1]	158	158	98%	1.00E-46	51.32	WP_162447161.1
GNAT family N-acetyltransferase [Rummeliibacillus pycnus]	157	157	92%	3.00E-46	56.34	WP_102691913.1
GNAT family N-acetyltransferase [Sporosarcina luteola]	157	157	91%	4.00E-46	58.16	WP_174842636.1
GNAT family N-acetyltransferase [Virgibacillus sp. Marseille-Q1616]	157	157	97%	4.00E-46	53.33	WP_164669852.1
**Aminoglycoside 6'-N-acetyltransferase I [Rhabdobacter roseus]**	157	157	98%	4.00E-46	50.33	MBB5285266.1
GNAT family N-acetyltransferase [Bacillus mediterraneensis]	157	157	91%	5.00E-46	56.03	WP_071460797.1
GNAT family N-acetyltransferase [Paenibacillus phyllosphaerae]	157	157	92%	5.00E-46	55.94	WP_183598596.1
GNAT family N-acetyltransferase [Rhabdobacter roseus]	157	157	87%	6.00E-46	52.59	WP_184175798.1

Supplementary Table 2. Top 10 DALI results using the PDB 6AO7 in this study. The most closely related structures, 1y9k and 1yvk, exhibit rmsd scores of less than 1 and 2 Å respectively. Both structures remain unpublished, and despite the low sequence identity, these structures exhibit the same dimeric structure as reported in this study (see also Figure 5).

**PDB id**	**Z score**	**rmsd**	**n_align**	**n_res**	**%id**
**6ao7**	**32.0**	**0**	**153**	**153**	**100**
**1y9k**	**26.8**	**0.9**	**149**	**154**	**48**
**1yvk**	**24.9**	**1.4**	**150**	**152**	**49**
2cnm	18.1	2.2	138	151	19
6wfn	17.9	2.3	142	151	18
4pv6	17.7	2.1	140	147	17
6wfg	17.7	2.3	141	150	18
6yga	17.6	2.2	140	159	20
4x5k	17.5	2.1	140	153	19
2ob0	17.5	2.3	141	154	18
